# The impact of underrepresented minority or marginalized identity status on training outcomes of MD-PhD students

**DOI:** 10.1186/s12909-023-04399-7

**Published:** 2023-06-08

**Authors:** Manuel A. Torres Acosta, Sidhanth Chandra, Sophia Li, Esther Yoon, Daniel Selgrade, Jeanne Quinn, Hossein Ardehali

**Affiliations:** 1 Northwestern University’s Medical Scientist Training Program, Northwestern University Feinberg School of Medicine, SQBRC 8-521, 303 E Superior Ave, Chicago, IL 60611 USA; 2 Northwestern University’s Department of Medicine, Northwestern University Feinberg School of Medicine, Chicago, IL 60611 USA; 3 The Ken and Ruth Davee Department of Neurology, Northwestern University Feinberg School of Medicine, Chicago, IL 60611 USA; 4 Northwestern University’s Department of Biomedical Engineering, Northwestern University Feinberg School of Medicine, Chicago, IL 60611 USA; 5 Northwestern University’s Center for Genetic Medicine, Northwestern University Feinberg School of Medicine, Chicago, IL 60611 USA

**Keywords:** Dual-degree training, MD-PhD, Physician scientist training, Diversity and inclusion

## Abstract

Dual-degree MD-PhD programs have historically lacked diversity of race, ethnicity, gender, sexual orientation, and other facets of identity. Like MD- and PhD-granting programs, MD-PhD program training environments are also marked by structural barriers that negatively impact measurable academic outcomes of underrepresented and/or marginalized students in academic medicine (racial and ethnic minority groups considered underrepresented by the National Institute of Health, sexual and gender minorities, individuals with disabilities, and individuals of low socioeconomic status). In this article, we review the existing literature on MD-PhD program disparities affecting students from these groups and provide recommendations grounded on the reviewed evidence. Our literature review identified four generalizable barriers that can impact the training outcomes of students from these marginalized and/or underrepresented groups: 1) discrimination and bias, 2) impostor syndrome and stereotype threat, 3) lack of identity-similar mentors, and 4) suboptimal institutional policies and procedures. We propose goal-oriented interventions that may begin to ameliorate the disparities present in MD-PhD program training environments that affect students from marginalized and/or underrepresented groups in academic medicine.

## Introduction

Physician scientists are key contributors to the advancement and bridging of medicine and biomedical research. Yet over the last thirty years, the percentage of physician scientists out of the total biomedical workforce has decreased, suggesting that the growth of the physician scientist workforce through a robust training pipeline has been stunted [1, 2]. The physician scientist workforce and dual-degree MD-PhD programs that train physician scientists have also historically lacked diversity of race, ethnicity, gender, sexual orientation, and other facets of identity [2, 3]. Importantly, increasing diversity in medicine and science is imperative to tackle inequities in access to and quality of care. Medical students from underrepresented racial and ethnic minority groups (Blacks or African Americans, Hispanics or Latinos, American Indians or Alaska Natives, Native Hawaiians, and Pacific Islanders), for example, report a higher commitment to the care of underserved populations, and African American patients are more likely to consider preventative interventions (e.g., vaccination) when counseled by a race-concordant doctor [4–7]. Similarly, diversity in science promotes innovation and better performance in problem solving, as evidenced by studies that have found that racially and ethnically diverse research teams are more scientifically productive as measured by increased numbers of publications, higher journal impact factors and a higher number of citations, compared to homogenous groups [8–10]. To ensure a more diverse physician scientist workforce that is able to meet the needs of a diverse population, it is necessary that dual-degree MD-PhD-granting programs not only recruit, but also retain trainees from diverse backgrounds historically marginalized and/or underrepresented in the workforce [11]. The training environments of these programs, however, continue to be burdened by structural barriers that hinder the advancement of these trainees, as exemplified by higher attrition rates from MD-PhD programs of underrepresented racial and ethnic minorities relative to white males [12]. Furthermore, certain marginalized trainees in MD-PhD programs may not benefit fully from diversity and inclusion programming because of their exclusion from groups classified as underrepresented by key institutions. For example, the National Institute of Health (NIH) includes individuals with disabilities but omits sexual and gender minorities when describing populations in need of more administrative support in academia based on their identity (see NIH notice NOT-OD-20–031). While sexual and gender minorities are not considered underrepresented by the NIH, their training experience can be negatively impacted by their marginalized identity, and the lack of their acknowledgement in NIH communications relating to increasing diversity in academia undermines the role intersectionality plays in exacerbating structural barriers for people from underrepresented groups that also have a marginalized identity. The NIH’s terminology stands in contrast to the one put forth by the Association of American Medical Colleges (AAMC), which defines "Underrepresented in medicine” as “those racial and ethnic populations that are underrepresented in the medical profession relative to their numbers in the general population" along with the separate designation of “Unique Populations” to define sexual and gender minorities as well as trainees with disabilities [13]. Despite the discordant definitions offered by these institutions, MD-PhD trainees from underrepresented minority groups and those with marginalized identities still deserve unique consideration in the efforts to promote a more diverse healthcare workforce. The declining numbers of physician scientists, the scarcity of underrepresented minorities in this profession, and the undeniable marginalization that students from certain communities experience calls for an evaluation of the physician scientist training in order to promote the retention and success of these students throughout the training process [14].

In this article, we provide an overview of the existing literature and propose solutions on MD-PhD program disparities affecting underrepresented racial and ethnic minorities (Blacks/African Americans, Native Hawaiians/Pacific Islanders, American Indians/Alaska Natives, and Hispanics/Latinos), sexual and gender minorities (SGMs, including non-heterosexual and non-cisgender individuals), individuals with disabilities, and individuals of low socioeconomic status (SES) or financially disadvantaged background. Collectively, these individuals will be referred to as Underrepresented and/or Marginalized students in Academic medicine (UrMAs).

Our review is divided into three sections. First, we assess the current state of UrMA training in MD-PhD programs. Second, we discuss four major barriers to successful MD-PhD training of UrMAs. For each barrier, we provide a collection of evidence of how the different underrepresented and/or marginalized groups composing the UrMA umbrella term are affected by that barrier. Lastly, we discuss a selection of published solutions and provide recommendations informed by the literature aimed at addressing these disparities. Peer-reviewed publications that specifically address current trends and outcomes of MD-PhD UrMAs are scarce; therefore, as MD-PhD trainees are de facto participants of curriculums prepared by MD-only and PhD-only programs at all points in their training, our review is supplemented with publications on MD-only, PhD-only, and residency programs. The goal of this review is to support future efforts of MD-PhD programs to address disparities affecting UrMAs pursuing a physician scientist career, and to invite readers and relevant institutions to consider how the intersection of different identities contained in the UrMA umbrella term may exacerbate the identified disparities.

### Current state of underrepresented and/or marginalized student training in MD-PhD programs

Dual-degree training via MD-PhD programs has been established since the 1950s. MD-PhD programs are either partially funded by the NIH through T32 awards from the National Institute of General Medical Sciences and known as a Medical Scientist Training Programs (MSTPs, first launched in 1964) or funded entirely at the individual institutional level through research grants, fellowships, and institutional commitments [15]. There is also the NIH MD-PhD Partnership Training Program, which partners with academic universities to support the PhD portion of MD-PhD training in MD-granting-only institutions. The AAMC currently lists 122 MD-PhD programs in the US across 43 states and Washington D.C., with more programs concentrated in New York, California, and Illinois and at least 5000 MD-PhD students in training [15, 16]. Of these programs, there are 50 MSTPs supporting around 1000 trainees [17]. There is limited publicly available data on MD-PhD program applicants and matriculation per institution, so it is unclear which programs have higher percentages of matriculants from certain UrMA groups (e.g., SGMs). Of note, there are currently 6 Historically Black Colleges or Universities (HBCUs) with medical schools, which play an outsized role in the education of Black/African American medical students [18, 19]. Of these, three HBCUs (Meharry, Howard, and Morehouse) have MD-PhD programs, making up approximately 2.4% of all MD-PhD programs.

Most MD-PhD programs consist of three training phases and have an estimated completion length of 7–9 years [20, 21]. During the *pre-clinical phase*, students partake in the didactic portion of the medical school curriculum. This first phase typically concludes with the first US Medical Licensing Exam (USMLE 1), after which they begin the *graduate phase* of their training, following selection of their thesis mentor. During this second phase, MD-PhD students are required to meet certain milestones for graduation that can include: the publication of a first-authored, peer-reviewed research article, the successful composition and defense of a graduate thesis and a mock research proposal (i.e., “qualifying exam”), and the presentation of original research in an academic setting. After obtaining their doctoral degree, students move on to clerkship rotations as part of the *clinical phase* of their training. This last phase has students identifying clinical areas of interest to pursue during their residency training, acquiring advanced clinical knowledge and skills required to pass the US Medical Licensing Exam 2 (USMLE 2), and partaking in residency program interviews.

The overall attrition rate is estimated to be 3% for both MD-PhD and MD-only students. This rate remains well below the 31–50% range estimated for PhD programs in engineering, life sciences, social sciences, mathematics, and physical sciences, suggesting that a commitment to clinical training influences trainee attrition [22]. However, around 75% of non-graduating MD-PhD students cite “non-academic” reasons for attrition, while only around 50% of non-graduating MD-only students cite these same reasons [23]. This discrepancy between MD-PhD and MD-only students may be attributed to factors that are specific to the dual-degree training curriculum, such as the longer time-to-degree, the difficult transitions between pre-clinical, graduate, and clinical phases of the training, among others [24]. MD-PhD students belonging to the UrMA umbrella term face both shared and subgroup-specific challenges during their training that may contribute to a significantly higher attrition rate or lower quality of life compared with non UrMAs; for example, MD-PhD students from underrepresented racial and ethnic minority groups have a higher attrition rate relative to white MD-PhD students [12]. Some UrMA subgroups affected by structural barriers during MD-PhD training are outlined below.

**Fig. 1:**
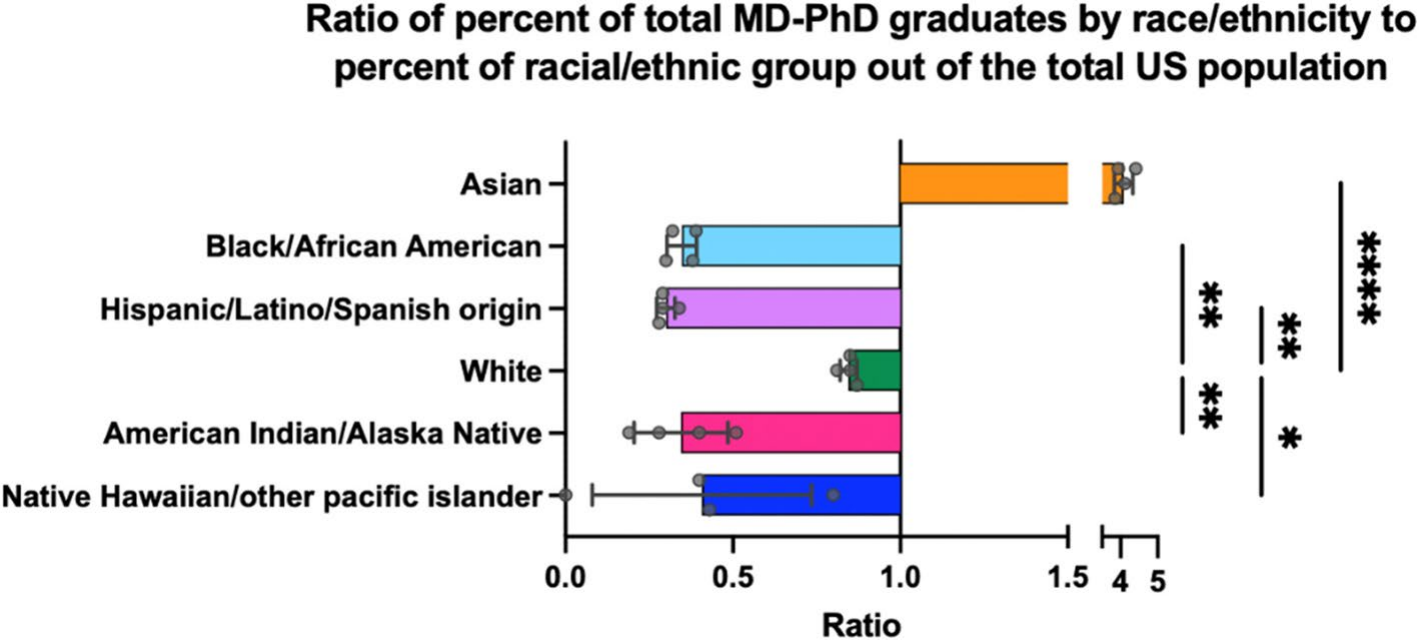
Ratio of percent (%) of total MD-PhD graduates by race or ethnicity to % of racial or ethnic group’s fraction out of the total US population. Demographic data of MD-PhD graduates from academic years 2016–2017 to 2019–2020 was obtained from AAMC’s website (AAMC data table B-13). Demographic data of the US population was obtained from the 2020 US census website. The averages of the ratios of graduating MD-PhD students to the percentage of the corresponding racial and ethnic category from the total US population were compared using a one-way analysis of variance (ANOVA); * = *p*-val < .05, ** = *p*-val < .01, **** = *p*-val < .0001

Underrepresented racial and ethnic minorities constituted around 11% of all MD-PhD graduates from 2016 to 2020 [3]. These individuals remain underrepresented in the MD-PhD graduate pool relative to their respective community’s contribution to the total US population. For example, African American MD-PhD graduates corresponded to around 5% of all MD-PhD graduating students during 2016–2020, meaning that African Americans make up less than half of the expected MD-PhD graduates if graduating classes were to recapitulate the demographics of the general US population; similar trends can be observed for other racial and ethnic groups (Fig. 1) [23, 25]. Moreover, underrepresented racial and ethnic minorities encounter barriers to performance in key academic parameters important for admission into post-graduate training, including USMLE 1 and 2 scores and total number of first-author publications during graduate school [26, 27]. Women constituted approximately 35% of all graduating MD-PhD students between 2005–2014, and although this represents an 8% increase in female graduates compared to the previous decade, women still remain underrepresented in dual-degree programs and also publish less on average during graduate school than their male counterparts [20, 27]. SGM students also face their own set of structural barriers throughout their training. One study of medical students that matriculated in 2010 found that lesbian, gay and bisexual (LGB) students constituted 5% of studied matriculants, although this is likely an underestimation of the representation of these students given the barriers associated with self-disclosure of SGM identity; data on the representation of non-cisgender individuals in dual-degree programs is not available [28–30]. Students with disabilities may also have higher attrition rates from medical school relative to medical students without protected disabilities according to the limited data available [31]. A survey administered to medical school disability services administrators found that 2.7% of the students from responding institutions reported having a disability [32]. This survey found the following prevalence among respondents who disclosed having a disability: ADHD (33.7%), learning disabilities (21.5%), psychological disabilities (20.0%) and mobility disabilities (2.5%).

### Barriers to successful training of underrepresented and/or marginalized students in MD-PhD programs

#### Bias, microaggressions and discriminatory attitudes in the training environment

Constant exposure to an unsafe academic environment, particularly in the form of microaggressions and discrimination, contributes to the poor well-being of underrepresented racial and ethnic minorities and thereby impacts their performance and retention [33]. Survey data of MD-PhD and MD-only students indicates that underrepresented racial and ethnic minorities are significantly more likely to have faced discrimination on the basis of race and gender relative to their white colleagues, and that their degree of burnout and disengagement is correlated with the extent to which they experience discrimination [33–36]. Unfortunately, underrepresented racial and ethnic minorities sometimes encounter these attitudes within their own graduate programs and research groups during their PhD training as well [37, 38]. Underrepresented racial and ethnic minorities are also less likely to be inducted to the Alpha Omega Alpha (AΩA) honor medical society relative to white males, even when controlling for USMLE 1 scores, which can impact their competitiveness for selection into post-graduate physician scientist training programs [39]. Data on the experiences of LGB medical students indicate they are also subjected to discrimination, and that many report they do not feel comfortable disclosing their sexual orientation in their training environment [35, 40, 41]. A survey on transgender and nonbinary medical students and physicians found that half of respondents had yet to disclose their non-cisgender status to their medical school, citing fear of transphobia and/or discrimination/harassment as reasons for non-reporting [42]. Seventy-eight percent of all survey participants (of which 58% were medical students) also reported censoring speech and/or mannerisms in their training environment; transgender graduate students also report similar experiences during their PhD training [43]. Faculty members are not the only source of SGM discrimination; in fact, a survey of medical students at the University of Ottawa found that the majority of discrimination experienced by SGM students originated from fellow medical students [44]. Moreover, female pre-clinical medical students are more likely to report experiencing, observing, or hearing about at least one incident of gender discrimination and sexual harassment during medical school relative to males [45]. Importantly, studies suggest that medical students who identify with two or more underrepresented or marginalized identities experience higher incidences of discrimination and bias, consistent with the literature on intersectionality as a predictor of the magnitude and frequency of negative experiences related to one’s identities [36, 46].

#### Disproportionate burden of non-academic stressors

According to a 2014 survey administered by the AAMC to second-year medical students, many UrMA subgroups (underrepresented racial and ethnic minorities, LGB students, and first-generation college students) experience either significantly higher levels of stress, fatigue, financial concerns, significantly lower quality of life, social support, or a combination of these when compared to white cis-gender heterosexual individuals [47]. Another survey-based study assessing the barriers to successful residency training anticipated by MD-PhD trainees identified both general and specific concerns held by individuals from underrepresented racial and ethnic groups [34]. Hispanic and Black MD-PhD students reported that the caretaking of others and being financially responsible for individuals other than themselves were considerable, non-work-related responsibilities they foresee themselves facing during residency. These specific concerns were significantly higher for these underrepresented racial and ethnic minorities when compared to white males. While these data represent anticipated challenges for residency and not dual-degree training, they do provide insight into some of the social pressures students from underrepresented racial and ethnic minority groups are more likely to face throughout their academic career. Other studies have found that LGB medical students report higher levels of depression, lower levels of perceived social support, and discomfort with disclosure of their sexuality in clinical settings [28, 40, 41]. Importantly, data suggests that differences in USMLE1/2 performance seen with individuals from underrepresented racial and ethnic minority groups are explained in part by this disproportionate burden of non-academic stressors [26].

#### Lack of identity-concordant mentors and the negative implications of academic medicine’s “hidden curriculum”

Mentorship of women and of individuals from underrepresented racial and ethnic minority groups by faculty members that they can identify with benefits and facilitates the success of these students in academic medicine and other higher education settings [48–51]. However, recruitment of faculty from underrepresented racial and ethnic minority communities to medical schools has stalled over the past three decades [52, 53]. So, while most MD-PhD programs do offer mentorship networks for their students to partake in, the demographics of participating mentors likely recapitulate the demographics of the respective institution’s faculty, who are predominantly able-bodied, cis-gender, heterosexual white males [54]. Irrespective of the lack of diversity present in institutional mentorship networks, individuals from underrepresented racial and ethnic minority groups are less likely to receive formal mentorship relative to white males throughout their career [55]. Moreover, SGM trainees that report having negative experiences as a consequence of their identity during their medical training cited a lack of non-cisgender mentors as a contributing factor to their negative experiences [42]. The absence of a diverse mentorship network exacerbates the negative implications of the “hidden curriculum” of academic medicine, which refers to the lessons and expectations (e.g., the importance of networking for success in academia) that are embedded in the organizational structure and culture of academic medicine but are not explicitly taught or communicated through the curriculum [50, 56, 57]. The power of the hidden curriculum is exemplified by the fact that tenure-track faculty are 25 times more likely than others to have a parent with a PhD [58, 59]. These data imply that first-generation students, who are more likely to lack previous exposure to the innerworkings of academia and are more likely to be of an underrepresented racial and ethnic minority group, are less likely to receive future tenure and therefore less likely to succeed in academic medicine [59].

#### Impostor syndrome and stereotype threat

A survey assessing medical student trainee attitudes toward careers in academic medicine found that the Black/African American and Hispanic/Latino survey respondents were significantly more in agreement with the claim that underrepresented racial and ethnic minority groups have a harder time succeeding in academic medicine relative to white males [60]. This attitude likely reflects both their perception of real barriers that would impede their success in academia, but also the prevalence of impostor syndrome in these trainee groups. Female medical students are also more likely to experience imposter syndrome than male counterparts [61]. Students from underrepresented racial and ethnic minority groups pursuing a healthcare-related career are also more likely to report feeling anxious about their performance and believe this anxiety has negatively impacted their performance; indeed, these students are more likely to attribute anxiety about their performance to negative stereotypes about the group with which they identify [62]. Accordingly, impostor syndrome and stereotype threat have been shown to disproportionately affect black students and their performance, but whether this holds true for other UrMA subgroups remains to be determined [63].

#### Lack of supportive institutional policies

Trainees in academic medicine who aspire to bear and rear children can encounter poor institutional support for this aspiration, and this burden falls disproportionately on women and SGM trainees. For example, survey data indicates that many residency program directors perceive maternity leave to have more of a negative impact on female trainee’s time-to-board certification and fellowship opportunities than paternity leave does on male trainees [64]. Poor support for childbearing and rearing also come in the form of single-gender policies during the graduate phase of training that denies paid paternity leave by dual-degree programs [65]. These and many other factors shift the brunt of childbearing to female trainees and may contribute to higher attrition rates for women in certain MD-PhD programs. Another example pertains to programs’ support of individuals with protected disabilities. A substantial portion of MD-granting programs expect students with disabilities to provide their own accommodations or lack clear technical standards outlining which party (the student or the institution) holds the locus of responsibility in this regard [66]. This lack of clear institutional policies pertaining to accommodations for individuals with protected disabilities can contribute to their attrition and hinder their performance.

### Solutions to ameliorate the attrition and barriers to performance of underrepresented and/or marginalized students in MD-PhD programs

#### Diversify the academic mentorship network available to MD-PhD students

As discussed, mentorship of dual-degree trainees from underrepresented racial and ethnic groups by faculty members that they identify with can facilitate these students’ success. Yet, these faculty members tend to be saturated by administrative responsibilities meant to promote institutional diversity and inclusion and therefore may be unable to undertake mentorship responsibilities. This phenomenon has been coined the “ambassador role” or “minority tax”, and many faculty members from underrepresented racial and ethnic groups report that this can undermine their own professional goals and responsibilities [67, 68]. Dual-degree programs can ameliorate the burden placed on existing faculty members by recruiting physician scientists from underrepresented and/or marginalized groups in academic medicine as program administrators, who interact with dual-degree trainees frequently. Moreover, if faculty are expected to mentor a disproportionate number of trainees because of their role in mentoring identity-concordant students, then they must also be rewarded properly for their work (both through remuneration and career advancement) and their other administrative responsibilities must be reduced accordingly so as to prevent the hindering of career progress by their mentorship responsibilities. Training in culturally-aware mentorship for faculty may also reduce biased mentoring practices, change approaches toward mentee and colleague interactions, and lighten the mentorship load for faculty over-extended by their “minority tax”. MD-PhD programs can also sponsor the participation of UrMAs to identity-specific research conferences, such as the Annual Biomedical Research Conference for Minoritized Scientists (ABRCMS), the Society for the Advancement of Chicanos/Hispanics and Native Americans in Science’s (SACNAS) annual conference, and the National LGBTQ Health Conference, where UrMAs have access to a more diverse network of mentors.

#### Focus administrative support and initiatives on helping students achieve the academic milestones needed to advance MD-PhD training

As discussed, the transition between the different phases of dual-degree training (and the academic milestones required for each) come with a set of challenges frequently cited by non-graduating students that may explain some of the attrition and barriers to performance of trainees from underrepresented racial and ethnic groups [24]. While a diverse mentorship network can provide substantial support throughout these transitions, enrolled UrMA students who have successfully transitioned between these phases can also play a key role in assisting identity-concordant students that have yet to transition between phases. Therefore, it is quintessential for MD-PhD programs to provide space in their curriculum for peer-to-peer and near-peer mentorship in the form of student Q&A panels that address all program phase transitions, student groups that support transitioning students, as well as social activities that foster community building and conversations about the challenges that come with dual -degree training and strategies to overcome them. For these initiatives to be successful, programs must be willing to commit a significant portion of their budget to offer positive incentives for participation, such as providing food or leisure activities. Various PhD-granting programs have implemented initiatives like these with great success. For example, the University of Texas MD Anderson Cancer Center UT Health Graduate School of Biomedical Sciences created student groups specifically tailored to support students from underrepresented racial and ethnic minority groups throughout their training, which yielded an improvement in their candidacy exam scores and retention rates over time [69]. Notably, while including UrMA peer mentors in these spaces is ideal, program administrators should provide appropriate compensation for their engagement or be careful not to place excessive burden on successful UrMA students to mentor identity-concordant trainees at lower stages of training, for just like faculty members, trainees are also susceptible to the “minority tax” phenomenon [70, 71].

#### Facilitate access to professional opportunities meant for students of underrepresented and/or marginalized identity

To address performance barriers experienced by UrMA dual-degree students, program administrators must leverage existing opportunities to enhance UrMA students’ training and facilitate UrMA engagement with them. For example, myriad institutions and organizations offer grants specifically geared towards subsets of UrMA students, such as the NIH’s Ruth L. Kirschstein National Research Service Award *Individual Predoctoral Fellowship to Promote Diversity in Health-Related Research*, among others. The NIH also offers “Diversity Supplements” for select funding mechanisms, including the R series and P series awards. These supplements represent additional funding opportunities for the training of students the NIH considers underrepresented in the extramural scientific workforce. Communicating these opportunities to the relevant students is necessary but not sufficient; dual-degree programs should also provide formal guidance on how to apply for these grants, including how to properly address the sections pertaining to diversity and inclusion that are required to receive these awards. MD-PhD programs can also leverage the opportunities for UrMA training enhancement offered by local, identity-based, and student-led organizations that belong to either the medical or graduate schools, including, for example, chapters of the Latino Medical Student Association, “Out Lists” meant to make SGM faculty institutionally visible, among others. Program administrators should proactively advertise the existence of these organizations and their professional development events to applicable UrMA students to not only achieve UrMA engagement with these opportunities, but also help them build community throughout their training [72].

#### Finalize the transition to a pass/fail (P/F) curriculum and standardized testing format

Standardized testing and pre-clinical training grades are not predictive of clinical performance, hence why USMLE 1 has moved towards a pass/fail grading system and many academic medicine institutions have followed suit with their own curriculum [26, 73]. Considering all the structural barriers that contribute to poor performance of students from underrepresented racial and ethnic minority groups in these areas, moving towards a P/F format for pre-clinical grades would alleviate some of the factors that limit the selection of some UrMAs who apply to post-graduate physician scientist training programs (PSTPs) and residency programs. P/F grading format may also alleviate feelings of impostor syndrome that students experience in the face of a suboptimal grade.

#### Create appropriate spaces for discussion and education of issues pertaining to individuals of underrepresented and/or marginalized identity

Many UrMA subgroups believe that the creation of spaces for discussion of UrMA issues would increase their sense of community and support [74–76]. For example, a survey administered by Stanford Medicine found that, when asked about which strategies would “improve the participants’ sense of SGM community”, SGM students gave overwhelming support to increased diversity, bias, and sensitivity training [77]. Various institutions have administered interventions meant to educate about topics pertaining to UrMA issues as part of their curriculum. The format of these interventions ranges from workshops (sharing of information by content experts) to faculty- or student-led group discussions (see Table 1 for examples of published interventions and their success in improving measured outcomes). These interventions are good opportunities to educate the student body about the identity-specific struggles faced by patients and fellow UrMA students when engaging with the healthcare system and academia, respectively. Importantly, we recommend that programs administer the pre- and post-intervention questionnaires of these published interventions, when available, to measure how the intervention is received by the program’s student body.

**Table 1:** Interventions that address diversity, equity, and inclusion topics along with their success in improving the outcomes measured

Topic addressed by interventions	Intervention	Outcomes measured pre-and post-intervention	Successful in improving measured outcomes? (*p*-val < .05)
**Impostor syndrome**	[78]	Confidence in recognizing imposter syndrome and taking action to address it	N/A
	[79]	Fraction of participants that would use information from impostor syndrome workshop in the future	N/A
**Academic appointment and promotion** (an example of the “hidden curriculum” of academic medicine)	[80]	Confidence in navigating tasks related to pursuing academic appointment	YES
	[81]	Extent to which workshop shared 1) key terminology associated with becoming an academic physician, 2) considerations a new physician should make before joining an academic physician practice, and 3) an optimal timeline for securing an academic physician position	YES
	[82]	Participant’s belief that a career in academic medicine would allow them to serve in a leadership role at a medical school	YES
**Identifying and communicating with prospective mentors**	[83]	Confidence in finding a mentor in academic medicine and having a successful relationship with them	YES
	[84]	Participant identification of strengths and weaknesses in current mentoring relationships	YES
**Diversity in academic medicine leadership: why it is important and how can URMs contribute to it**	[85]	Confidence in ability to: 1) define self-leadership, 2) identify leadership roles that align with own interests, 3) assess own self-leadership skills, and 4) effectively serve as leader at an academic institution given one’s identity	YES
**Racism, Discrimination, and Microaggressions**	[86]	Comfort discussing issues relating to race/ethnicity, gender identity/expression, sexual orientation, and spirituality with colleagues or trainees	N/A
	[87]	Confidence in 1) recognizing and interrupting microaggressions, and 2) in supporting peers when they experience microaggressions	N/A
	[88]	Knowledge about microaggressions, confidence in responding to microaggressions, and commitment to intervening when witnessing microaggressions	YES
**Promoting a safe space learning environment for LGBT medical students**	[89]	Confidence in 1) responding appropriately when an LGBT community member comes out, and 2) ability to ask community members about their preferred pronouns	YES

### Concluding remarks

**Fig. 2:**
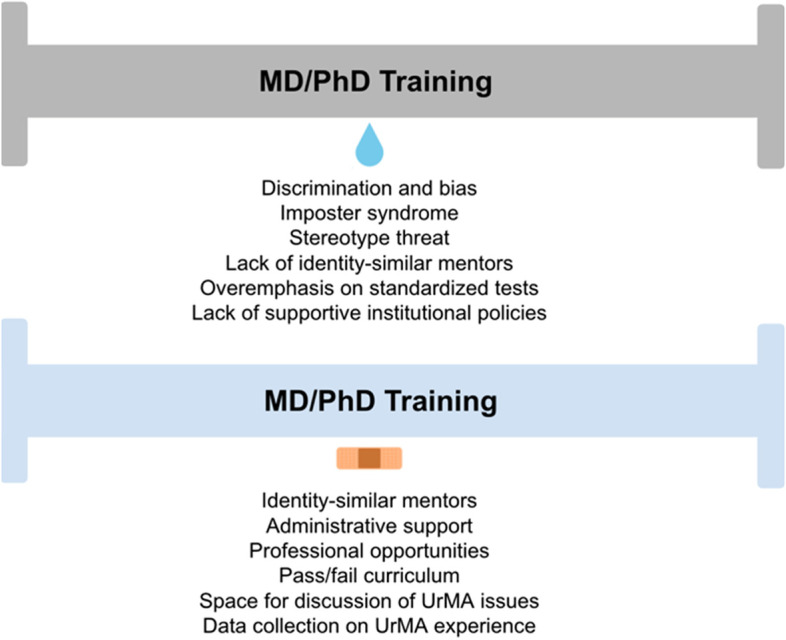
Summary of barriers affecting training outcomes of underrepresented and/or marginalized MD-PhD students along with proposed solutions

The goal of this literature review was to provide a resource for MD-PhD programs on how underrepresented and/or marginalized identity status may impact training outcomes and what evidence-based interventions could aid UrMA students during their training. To this end, we have summarized the barriers affecting training outcomes and quality of life of UrMAs pursuing the physician scientist career track according to data on MD-only, PhD-only, and MD-PhD students (Fig. 2). Because of the lack of published literature on MD-PhD programs, we chose to also include MD-only and PhD-only studies in our review. However, the literature included in this article does not explain all the observed UrMA disparities in the MD-PhD training pipeline. We specifically found a lack of data on how the intersection of different identities modifies these barriers, and data from underrepresented and/or marginalized individuals in other contexts, including most STEM careers, supports the contention that intersectionality can indeed exacerbate disparities [46, 90, 91]. We have also suggested goal-oriented and evidence-based interventions to address said barriers, although more data on the efficacy of other interventions and the specific issues they aim to address are needed. Importantly, most of these groups share the fact that they are *minoritized*, often as an unintended consequence of systems established from historical legacy [92]. Without the appropriate interventions, these individuals will continue to be minoritized by the current physician scientist training pipeline. As the predominant cradle of the next generation of physician scientists, it is the responsibility of dual-degree MD-PhD programs to leverage evidence-based interventions to modernize their training practices and to provide equitable training opportunities for UrMAs.

## Data Availability

Not applicable.

## References

[1] Feldman AM (2014). The National Institutes of Health Physician-Scientist Workforce Working Group report: a roadmap for preserving the physician-scientist. Clin Transl Sci.

[2] National Institute of Health. Physician-scientist workforce working group report. 2014. https://acd.od.nih.gov/documents/reports/PSW_Report_ACD_06042014.pdf. Accessed 10 May 2022. 10.1111/cts.12209 PMC5439807 25123835

[3] AAMC: Table B13: Race/Ethnicity Responses (Alone and In Combination) of MD‐PhD Graduates of U.S. MD‐Granting Medical Schools, 2016‐2017 through 2020‐2021. 2021.

[4] Alsan M, Garrick O, Graziani G (2019). Does Diversity Matter for Health? Experimental Evidence from Oakland. American Economic Review.

[5] Garcia AN, Kuo T, Arangua L, Perez-Stable EJ (2018). Factors Associated With Medical School Graduates' Intention to Work With Underserved Populations: Policy Implications for Advancing Workforce Diversity. Acad Med.

[6] Bailey JA, Willies-Jacobo LJ (2012). Are disadvantaged and underrepresented minority applicants more likely to apply to the program in medical education-health equity?. Acad Med.

[7] Takeshita J, Wang S, Loren AW, Mitra N, Shults J, Shin DB, Sawinski DL (2020). Association of Racial/Ethnic and Gender Concordance Between Patients and Physicians With Patient Experience Ratings. JAMA Netw Open.

[8] Freeman RB, Huang W. Collaborating with people like me: ethnic co-authorship within the US. NBER Working Paper No. 19905. 2014.

[9] Campbell LG, Mehtani S, Dozier ME, Rinehart J (2013). Gender-heterogeneous working groups produce higher quality science. PLoS ONE.

[10] Page SE (2017). The diversity bonus: how great teams pay off in the knowledge economy.

[11] Green M, Wayne DB, Neilson EG (2019). Medical Education 2020-Charting a Path Forward. JAMA.

[12] Jeffe DB, Andriole DA, Wathington HD, Tai RH (2014). Educational outcomes for students enrolled in MD-PhD programs at medical school matriculation, 1995–2000: a national cohort study. Acad Med.

[13] Ogunyemi D. Unique populations. https://www.aamc.org/professional-development/affinity-groups/gfa/unique-populations. Accessed 17 Apr 2023.

[14] Blish CA. Maintaining a Robust Pipeline of Future Physician-Scientists. J Infect Dis. 2018;218(suppl_1):S40-S43. 10.1093/infdis/jiy093 PMC6093428 30124975

[15] Harding CV, Akabas MH, Andersen OS (2017). History and Outcomes of 50 Years of Physician-Scientist Training in Medical Scientist Training Programs. Acad Med.

[16] MD-PhD degree programs by state. https://students-residents.aamc.org/applying-md/phd-programs/md-phd-degree-programs-state. Accessed 17 Apr 2023.

[17] NIGMS. Medical scientist training program. https://nigms.nih.gov/training/instpredoc/Pages/.PredocOverview-MSTP.aspx. Accessed 16 Mar 2023.

[18] Steele D. Will more medical schools mean more black doctors? https://www.insidehighered.com/news/2022/05/13/new-med-schools-planned-need-black-doctors-continues. Accessed 16 Mar 2023.

[19] Rodriguez JE, Lopez IA, Campbell KM, Dutton M (2017). The Role of Historically Black College and University Medical Schools in Academic Medicine. J Health Care Poor Underserved.

[20] Akabas MH, Brass LF. The national MD-PhD program outcomes study: outcomes variation by sex, race, and ethnicity. JCI Insight. 2019;4(19):e133009. 10.1172/jci.insight.133010 PMC6795407 31578303

[21] Brass LF (2018). Is an MD/PhD program right for me? Advice on becoming a physician-scientist. Mol Biol Cell.

[22] Young SN, VanWye WR, Schafer MA, Robertson TA, Poore AV (2019). Factors Affecting PhD Student Success. Int J Exerc Sci.

[23] AAMC. Graduation rates and attrition rates of U.S. medical students. https://www.aamc.org/data-reports/students-residents/report/graduation-rates-and-attrition-rates-us-medical-students. Accessed 18 Jan 2022.

[24] Chakraverty D, Jeffe DB, Dabney KP, Tai RH. Exploring Reasons That U.S. Md-Phd Students Enter and Leave Their Dual-Degree Programs. Int J Dr Stud. 2020;15:461–483. 10.28945/4622 PMC8018685 33815015

[25] US Census Bureu. DP05 2019: American community survey 1-year estimates data profiles. 2020. https://data.census.gov/cedsci/table?q=race%20and%20ethnicity&tid=ACSDP1Y2019.DP05. Accessed 1 June 2022.

[26] Rubright JD, Jodoin M, Barone MA (2019). Examining Demographics, Prior Academic Performance, and United States Medical Licensing Examination Scores. Acad Med.

[27] Roksa J, Wang Y, Feldon D, Ericson M (2022). Who is publishing journal articles during graduate school? Racial and gender inequalities in biological sciences over time. Journal of Diversity in Higher Education.

[28] Przedworski JM, Dovidio JF, Hardeman RR, Phelan SM, Burke SE, Ruben MA, Perry SP, Burgess DJ, Nelson DB, Yeazel MW (2015). A Comparison of the Mental Health and Well-Being of Sexual Minority and Heterosexual First-Year Medical Students: A Report From the Medical Student CHANGE Study. Acad Med.

[29] Mansh M, White W, Gee-Tong L, Lunn MR, Obedin-Maliver J, Stewart L, Goldsmith E, Brenman S, Tran E, Wells M (2015). Sexual and gender minority identity disclosure during undergraduate medical education: "in the closet" in medical school. Acad Med.

[30] Madrigal J, Rudasill S, Tran Z, Bergman J, Benharash P (2021). Sexual and gender minority identity in undergraduate medical education: Impact on experience and career trajectory. PLoS ONE.

[31] Teherani A, Papadakis MA (2013). Clinical performance of medical students with protected disabilities. JAMA.

[32] Meeks LM, Herzer KR (2016). Prevalence of Self-disclosed Disability Among Medical Students in US Allopathic Medical Schools. JAMA.

[33] Perry SP, Hardeman R, Burke SE, Cunningham B, Burgess DJ, van Ryn M (2016). The Impact of Everyday Discrimination and Racial Identity Centrality on African American Medical Student Well-Being: a Report from the Medical Student CHANGE Study. J Racial Ethn Health Disparities.

[34] Siebert AL, Chou S, Toubat O, Adami AJ, Kim H, Daye D, Kwan JM (2020). Factors associated with underrepresented minority physician scientist trainee career choices. BMC Med Educ.

[35] Hill KA, Samuels EA, Gross CP, Desai MM, Sitkin Zelin N, Latimore D, Huot SJ, Cramer LD, Wong AH, Boatright D (2020). Assessment of the Prevalence of Medical Student Mistreatment by Sex, Race/Ethnicity, and Sexual Orientation. JAMA Intern Med.

[36] Teshome BG, Desai MM, Gross CP, Hill KA, Li F, Samuels EA, Wong AH, Xu Y, Boatright DH (2022). Marginalized identities, mistreatment, discrimination, and burnout among US medical students: cross sectional survey and retrospective cohort study. BMJ.

[37] Rodriguez SL, Perez RJ, Schulz JM (2022). How STEM lab settings influence graduate school socialization and climate for students of color. Journal of Diversity in Higher Education.

[38] Alexander QR, Hermann MA (2016). African-American women’s experiences in graduate science, technology, engineering, and mathematics education at a predominantly white university: A qualitative investigation. Journal of Diversity in Higher Education.

[39] Boatright D, Ross D, O'Connor P, Moore E, Nunez-Smith M (2017). Racial Disparities in Medical Student Membership in the Alpha Omega Alpha Honor Society. JAMA Intern Med.

[40] Lapinski J, Sexton P (2014). Still in the closet: the invisible minority in medical education. BMC Med Educ.

[41] Chester SD, Ehrenfeld JM, Eckstrand KL (2014). Results of an Institutional LGBT Climate Survey at an Academic Medical Center. LGBT Health.

[42] Dimant OE, Cook TE, Greene RE, Radix AE (2019). Experiences of Transgender and Gender Nonbinary Medical Students and Physicians. Transgend Health.

[43] Goldberg AE, Kuvalanka K (2019). dickey l: Transgender graduate students’ experiences in higher education: A mixed-methods exploratory study. Journal of Diversity in Higher Education.

[44] Nama N, MacPherson P, Sampson M, McMillan HJ (2017). Medical students' perception of lesbian, gay, bisexual, and transgender (LGBT) discrimination in their learning environment and their self-reported comfort level for caring for LGBT patients: a survey study. Med Educ Online.

[45] Stratton TD, McLaughlin MAW, Florence M, Fosson SE, Nora LM (2005). Does Students’ Exposure to Gender Discrimination and Sexual Harassment in Medical School Affect Specialty Choice and Residency Program Selection?. Acad Med.

[46] Cech EA: The intersectional privilege of white able-bodied heterosexual men in STEM. Sci Adv. 2022; 8(24):eabo1558. 10.1126/sciadv.abo1558 PMC9200289 35704581

[47] Grbic D, Sondheimer H. Personal well-being among medical students: findings from an AAMC pilot survey. Analysis Brief. 2014;11. https://www.aamc.org/media/7561/download?attachment. Accessed 19 Jan 2022.

[48] DeCastro R, Sambuco D, Ubel PA, Stewart A, Jagsi R (2013). Mentor networks in academic medicine: moving beyond a dyadic conception of mentoring for junior faculty researchers. Acad Med.

[49] Carapinha R, Ortiz-Walters R, McCracken CM, Hill EV, Reede JY (2016). Variability in Women Faculty's Preferences Regarding Mentor Similarity: A Multi-Institution Study in Academic Medicine. Acad Med.

[50] Williams SN, Thakore BK, McGee R (2016). Career Coaches as a Source of Vicarious Learning for Racial and Ethnic Minority PhD Students in the Biomedical Sciences: A Qualitative Study. PLoS ONE.

[51] Santa-Ramirez S (2022). Sink or swim: The mentoring experiences of Latinx PhD students with faculty of color. Journal of Diversity in Higher Education.

[52] Odei BC, Jagsi R, Diaz DA, Addison D, Arnett A, Odei JB, Mitchell D (2021). Evaluation of Equitable Racial and Ethnic Representation Among Departmental Chairs in Academic Medicine, 1980–2019. JAMA Netw Open.

[53] Bennett CL, Ling AY (2021). Proportions of Faculty Self-identifying as Black or African American at US Medical Schools, 1990–2020. JAMA.

[54] Table 11: U.S. medical school faculty by sex, race, ethnicity, and rank. 2020. https://www.aamc.org/system/files/2021-01/2020Table11.pdf. Accessed 19 Jan 2022.

[55] Beech BM, Calles-Escandon J, Hairston KG, Langdon SE, Latham-Sadler BA, Bell RA (2013). Mentoring programs for underrepresented minority faculty in academic medical centers: a systematic review of the literature. Acad Med.

[56] Lehmann LS, Sulmasy LS, Desai S, Acp Ethics P, Human Rights C (2018). Hidden Curricula, Ethics, and Professionalism: Optimizing Clinical Learning Environments in Becoming and Being a Physician: A Position Paper of the American College of Physicians. Ann Intern Med.

[57] Gabel R: The Hidden Curriculum: First Generation Students at Legacy Universities: Princeton University Press; 2021.

[58] Morgan AC, LaBerge N, Larremore DB, Galesic M, Brand JE, Clauset A (2022). Socioeconomic roots of academic faculty. Nat Hum Behav..

[59] Romero R, Miotto K, Casillas A, Sanford J (2020). Understanding the Experiences of First-Generation Medical Students: Implications for a Diverse Physician Workforce. Acad Psychiatry..

[60] Sanchez JP, Peters L, Lee-Rey E, Strelnick H, Garrison G, Zhang K, Spencer D, Ortega G, Yehia B, Berlin A (2013). Racial and ethnic minority medical students' perceptions of and interest in careers in academic medicine. Acad Med..

[61] Rosenthal S, Schlussel Y, Yaden MB, DeSantis J, Trayes K, Pohl C, Hojat M (2021). Persistent Impostor Phenomenon Is Associated With Distress in Medical Students. Fam Med..

[62] Ackerman-Barger K, Valderama-Wallace C, Latimore D, Drake C (2016). Stereotype Threat Susceptibility Among Minority Health Professions Students. J Best Pract Health Prof Diversity..

[63] Wyatt TR, Rockich-Winston N, Taylor TR, White D (2020). What Does Context Have to Do With Anything? A Study of Professional Identity Formation in Physician-Trainees Considered Underrepresented in Medicine. Acad Med..

[64] Sharpe EE, Ku C, Malinzak EB, Kraus MB, Chandrabose R, Hartlage SEH, Hanson AC, Schulte PJ, Pearson ACS (2021). A cross-sectional survey study of United States residency program directors' perceptions of parental leave and pregnancy among anesthesiology trainees. Can J Anaesth..

[65] Powell K (2019). Babies on board - Why US scientist-mums need support in the early years of parenthood. Nature..

[66] Zazove P, Case B, Moreland C, Plegue MA, Hoekstra A, Ouellette A, Sen A, Fetters MDUS (2016). Medical Schools' Compliance With the Americans With Disabilities Act: Findings From a National Study. Acad Med..

[67] Osseo-Asare A, Balasuriya L, Huot SJ, Keene D, Berg D, Nunez-Smith M, Genao I, Latimore D, Boatright D. Minority Resident Physicians’ Views on the Role of Race/Ethnicity in Their Training Experiences in the Workplace. JAMA Netw Open. 2018;1(5):e182723. 10.1001/jamanetworkopen.2018.2723 PMC6324489 30646179

[68] Mahoney MR, Wilson E, Odom KL, Flowers L, Adler SR (2008). Minority faculty voices on diversity in academic medicine: perspectives from one school. Acad Med..

[69] Wilson MA, DePass A, Bean AJ. Institutional Interventions That Remove Barriers to Recruit and Retain Diverse Biomedical PhD Students. CBE Life Sci Educ. 2018;17(2):ar27. 10.1187/cbe.17-09-0210 PMC5998306 29749848

[70] Dickins K, Levinson D, Smith SG, Humphrey HJ (2013). The minority student voice at one medical school: lessons for all?. Acad Med..

[71] Perez RJ, Motshubi R, Rodriguez SL (2022). “We are a huge source of labor”: Exploring STEM Graduate Students’ Roles in Changing Departmental Climate. Rev Higher Educ..

[72] Nakae S, Martinez S, Juarez JJ, Beltran Sanchez C (2023). The Impacts of Engagement in the Latino Medical Student Association. Health Equity..

[73] McGaghie WC, Cohen ER, Wayne DB (2011). Are United States Medical Licensing Exam Step 1 and 2 scores valid measures for postgraduate medical residency selection decisions?. Acad Med..

[74] Lantz MM, Fix RL, Davis BL, Harrison LN, Oliver A, Crowell C, Mitchell AM, García JJ (2016). Grad students talk: Development and process of a student-led social justice initiative. J Diversity Higher Educ..

[75] Chu W, Hart MJ, Kirchner KN, Paton MJ, Black CJ (2022). Addressing race and diversity in graduate education: Practices from student activism. J Diversity Higher Educ..

[76] Hung R, McClendon J, Henderson A, Evans Y, Colquitt R, Saha S. Student perspectives on diversity and the cultural climate at a U.S. medical school. Acad Med. 2007;82(2):184-192. 10.1097/ACM.0b013e31802d936a 17264699

[77] Stanford Medicine’s Office of Faculty Development and Diversity. Report on needs of the sexual and gender minority community at Stanford Medicine. In: Stanford Medicine. 2018. https://med.stanford.edu/content/dam/sm/faculty-diversity/documents/Metrics/SGM-Report_2018_Final.pdf. Accessed 20 Jan 2022.

[78] Baumann N, Faulk C, Vanderlan J, Chen J, Bhayani RK. Small-Group Discussion Sessions on Imposter Syndrome. MedEdPORTAL. 2020;16:11004. 10.15766/mep_2374-8265.11004 PMC7666839 33204832

[79] Rivera N, Feldman EA, Augustin DA, Caceres W, Gans HA, Blankenburg R. Do I Belong Here? Confronting Imposter Syndrome at an Individual, Peer, and Institutional Level in Health Professionals. MedEdPORTAL. 2021;17:11166. 10.15766/mep_2374-8265.11166 PMC8257750 34277932

[80] Callahan EJ, Banks M, Medina J, Disbrow K, Soto-Greene M, Sanchez JP. Providing Diverse Trainees an Early and Transparent Introduction to Academic Appointment and Promotion Processes. MedEdPORTAL. 2017;13:10661. 10.15766/mep_2374-8265.10661 PMC6338160 30800861

[81] Mason BS, Landry A, Sanchez JP, Williams VN. How to Find an Academic Position After Residency: Who, What, When, Where, Why, and How. MedEdPORTAL. 2018;14:10727. 10.15766/mep_2374-8265.10727 PMC6342352 30800927

[82] Dickerman J, Sanchez JP, Portela-Martinez M, Roldan E. Leadership and Academic Medicine: Preparing Medical Students and Residents to Be Effective Leaders for the 21st Century. MedEdPORTAL. 2018;14:10677. 10.15766/mep_2374-8265.10677 PMC6342431 30800877

[83] Ortega G, Smith C, Pichardo MS, Ramirez A, Soto-Greene M, Sanchez JP. Preparing for an Academic Career: The Significance of Mentoring. MedEdPORTAL. 2018;14:10690. 10.15766/mep_2374-8265.10690 PMC6342371 30800890

[84] Christou H, Dookeran N, Haas A, Di Frances C, Emans SJ, Milstein ME, Kram KE, Seely EW. Establishing Effective Mentoring Networks: Rationale and Strategies. MedEdPORTAL. 2017;13:10571. 10.15766/mep_2374-8265.10571 PMC6338153 30800773

[85] Lucas R, Kothari P, Adams C, 3rd, Jones L, Williams VN, Sanchez JP. We are All Leaders: Introducing Self-Leadership Concepts Through the Lens of Improving Diversity in the Health Care Workforce. MedEdPORTAL. 2020;16:11011. 10.15766/mep_2374-8265.11011 PMC7666830 33204835

[86] Sotto-Santiago S, Mac J, Duncan F, Smith J. "I Didn't Know What to Say": Responding to Racism, Discrimination, and Microaggressions With the OWTFD Approach. MedEdPORTAL. 2020;16:10971. 10.15766/mep_2374-8265.10971 PMC7394349 32754635

[87] Acholonu RG, Cook TE, Roswell RO, Greene RE. Interrupting Microaggressions in Health Care Settings: A Guide for Teaching Medical Students. MedEdPORTAL. 2020;16:10969. 10.15766/mep_2374-8265.10969 PMC7394346 32754633

[88] Ackerman-Barger K, Jacobs NN, Orozco R, London M. Addressing Microaggressions in Academic Health: A Workshop for Inclusive Excellence. MedEdPORTAL. 2021;17:11103. 10.15766/mep_2374-8265.11103 PMC7880252 33598543

[89] Gacita A, Gargus E, Uchida T, Garcia P, Macken M, Seul L, Brucker J, Wayne DB. Introduction to Safe Space Training: Interactive Module for Promoting a Safe Space Learning Environment for LGBT Medical Students. MedEdPORTAL. 2017;13:10597. 10.15766/mep_2374-8265.10597 PMC6338189 30800799

[90] Tsouroufli M, Rees CE, Monrouxe LV, Sundaram V (2011). Gender, identities and intersectionality in medical education research. Med Educ.

[91] Lett E, Dowshen NL, Baker KE. Intersectionality and Health Inequities for Gender Minority Blacks in the U.S. Am J Prev Med. 2020;59(5):639-647. 10.1016/j.amepre.2020.04.013 PMC7577994 32792281

[92] Sotto-Santiago S (2019). Time to Reconsider the Word Minority in Academic Medicine. J Best Pract Health Prof Diversity..

